# Response to ‘Systematic reviews do not always capture context of real-world intervention programmes for childhood obesity’ by Wild et al., 2021 in *BMC Public Health*

**DOI:** 10.1186/s12889-021-10487-4

**Published:** 2021-03-15

**Authors:** Robyn Littlewood, Oliver J. Canfell, Jacqueline L. Walker

**Affiliations:** 1 Health and Wellbeing Queensland, Queensland Government, The State of Queensland, Milton, QLD Australia; 2 Faculty of Health and Behavioural Sciences, The University of Queensland, St Lucia, QLD Australia; 3 Centre for Health Services Research, Faculty of Medicine, The University of Queensland, Herston, QLD 4006 Australia; 4 UQ Business School, Faculty of Business, Economics and Law, The University of Queensland, St Lucia, QLD Australia; 5 Digital Health Cooperative Research Centre, Australian Government, Sydney, NSW Australia; 6 School of Human Movement and Nutrition Sciences, Faculty of Health and Behavioural Sciences, The University of Queensland, St Lucia, QLD Australia

**Keywords:** Oceanic ancestry group, Obesity, Systematic review, Intervention, Child, Adolescent

## Abstract

In a correspondence to BMC Public Health, Wild et al. respond to our systematic review that synthesised results of interventions to prevent or treat childhood obesity in Māori and Pacific Islanders. Our review included the *Whānau Pakari* study as one of six included studies – a multidisciplinary intervention for Māori children and adolescents living with obesity led by their research team. Our review suggested that future research can incorporate stronger co-design principles when designing culturally-tailored interventions to maximise cultural specificity, enhance engagement, facilitate program ownership and contribute to improved health and weight-related outcomes. We commend *Whānau Pakari* and the team of Wild et al. on their sustained commitment to addressing obesity in priority populations and agree that systematic reviews struggle to capture real-world context of interventions for complex diseases such as obesity. In this article, we respond sequentially to the comments made by Wild et al. and (1) clarify the scope of our review article (2) reiterate our commendation of mixed-methods approaches that capture real-world context (3) explain a referencing error that caused a misinterpretation of our results (4) clarify our interpretation of some *Whānau Pakari* characteristics (5) welcome partnership to facilitate shared learning with Wild et al.

## Main text

We are writing in response to the Correspondence article from Wild et al. regarding our recent review article, ‘Interventions to prevent or treat childhood obesity in Māori and Pacific Islanders: a systematic review’ [1], published in issue 20 (May 2020) of *BMC Public Health*. We would like to sincerely thank Wild and colleagues for their considered response to our article. This letter aims to sequentially respond to key points made by Wild et al. relating to our assessment of their *Whānau Pakari* trial [2] in our systematic review.

We would like to strongly commend the multidisciplinary obesity intervention - *Whānau Pakari* – and its holistic, culturally-considered approach to helping Māori achieve persistent healthy lifestyle change. It is difficult and complex to design, implement and evaluate real-world, long-term interventions for obesity, especially in priority populations such as Māori. Our research team has experience in co-designing such interventions -– *Healthier Together* is a co-designed, community program targeting childhood obesity in Māori and Pacific Islander children and families in Queensland, Australia [3].

We agree that the rigid methodology and scope of systematic reviews can fail to adequately capture the context of complex, pragmatic and multicomponent real-world interventions. Qualitative and mixed-methods research are invaluable in providing a patient-centred, evaluative lens to complex intervention trials. Results from this type of research are necessary to estimate qualitative trial effectiveness for indicators – e.g. family cohesion, enjoyment of engaging in health-related behaviours and self-confidence [4] – that are difficult to measure using traditional quantitative methods. This is especially true for complex interventions such as those required to address childhood obesity.

Our systematic review was a first step to synthesising the literature of interventions to prevent or treat childhood obesity in Māori and Pacific Islander children and families. Therefore, our scope focused on determining the effectiveness of these interventions on anthropometric, cardiometabolic, psychological and behavioural outcomes as components of health. The intent was to establish a foundational measure of effectiveness that can guide future decision-making when designing a childhood obesity intervention for Māori and Pacific Islander children and families.

We agree that a realist synthesis or meta-ethnography of qualitative research would have provided stronger contextual understanding of the interventions reported in our review – we see this as a logical step for future research. Realist syntheses for childhood obesity are emerging in the literature as a relatively new approach to intervention analysis [5, 6]. We were deliberate in describing and reporting the qualitative results of the mixed-methods study by Chansavang et al. [7], and commending its approach. The qualitative research cited by Wild et al. relating to *Whānau Pakari* provides invaluable, deeper insight into participant barriers and facilitators to engagement, and would have been included in our discussion but was published after the completion of our review.

Our statement that physical activity was the primary focus of *Whānau Pakari* was the unfortunate result of a referencing error – we apologise. The statement in question reports *“physical activity was the primary focus in 3 of 4 programs assessing anthropometric outcome measures”.* We mistakenly referenced all 4 programs (the 4th being *Whānau Pakari*) instead of only referencing the three programs that focused on physical activity. This assertion would have been inconsistent with our reporting of the *Whānau Pakari* trial throughout the review; we accurately described the multidisciplinary, multicomponent design of *Whānau Pakari* in Table 2 and ‘study characteristics’ section of our review.

We commend the evidence-based inclusion of multicomponent outcome measures (anthropometric, physical activity, psychological, behavioural) to assess the impact of *Whānau Pakari*. Dietary sessions including virtual supermarket tours, cooking sessions, portion size and the concept of healthy food were integrated into *Whānau Pakari* and acknowledged in Table 2 of our systematic review [1]; however, a dietary outcome measure was not reported in the trial results [2]. We could not comment on the independent or synergistic effect of dietary intervention as its impact was not measured in *Whānau Pakari*. Dietary outcome measures were included in the published study protocol [8] of *Whānau Pakari* but not reported in the intervention trial included in our systematic review.

In our systematic review, we state that significant consultation with Māori stakeholders was limited to the initial set-up phase of *Whānau Pakari* [1]. We thank the authors for clarification – in their letter – that consultation continued throughout the trial and is ongoing in *Whānau Pakari*, as also mentioned in the study protocol [8]. In describing the consultation process with Māori stakeholders in the study protocol, details are limited to the inception stage of *Whānau Pakari* – this informed our conclusion that consultation was strongest in the set-up phase of *Whānau Pakari*. We could not comment on the strength or impact of consultation after the set-up phase as details were not provided in the study protocol or trial.

We adopted a precise approach to reporting details of consultation with Māori and Pacific Islander peoples across all included studies. This was to assess alignment with evidence-based co-design principles and methodologies. In the context of our systematic review, we used ‘co-design’ to refer to participatory, experience-based design of interventions for childhood obesity that empowers its target population with shared and equal decision-making, ownership and involvement across all stages of intervention conception, design, implementation and evaluation [1, 9]. Our intention was not nor is to market co-design as a silver bullet but as an enabler to effective intervention design to improve acceptability, participation and outcomes.

We agree that upstream determinants of health, especially socioeconomic disparities, require significant equity-driven prevention investment and institutional transformation to ‘close the gap’. Co-designed, cultural-tailoring of mixed-methods interventions for childhood obesity in priority populations such as Māori and Pacific Islanders will help to improve intervention engagement and effectiveness, and provide an empirical platform for future realist syntheses to precisely guide evaluation, investment and interventions for obesity. Due to the inherent complexity of childhood obesity interventions for priority populations, we welcome and encourage a partnership model with established research leaders such as Wild et al. to facilitate shared learning and contextualise approaches to improving Māori and Pacific Islander health.

## Data Availability

Not applicable.
